# The pandemic legacy face mask: hidden barrier or insignificant factor in exercise performance? A randomized crossover trial

**DOI:** 10.1097/MD.0000000000050179

**Published:** 2026-08-14

**Authors:** Meryem Bektaş Karakuş, Eren Timurtaş, Aysel Yildiz Özer

**Affiliations:** a Department of Physiotherapy and Rehabilitation, Faculty of Health Sciences, Acibadem Mehmet Ali Aydinlar University, Istanbul, Turkey; b Department of Cardiopulmonary Physiotherapy and Rehabilitation, Institute of Health Sciences, Bezmialem Vakif University, Istanbul, Turkey; c Department of Physiotherapy and Rehabilitation, Faculty of Health Sciences, Marmara University, Istanbul, Turkey.

**Keywords:** dyspnea, exercise tolerance, masks, surgical mask, young adult

## Abstract

**Background:**

Medical masks serve as a barrier that prevents droplet transmission – the primary route of infection for many respiratory diseases. The effects of protective medical mask use on exercise performance and the factors influencing mask compliance are equivocal. This study aimed to examine the impact of Type I medical mask use on lower extremity endurance, exercise capacity, exercise-related parameters, and comfort levels in healthy young adults.

**Methods:**

In this randomized crossover study, participants completed the 30-second sit-to-stand test, the incremental shuttle walk test (ISWT), and the 6-minute walk test (6MWT) in random order, both with and without a mask. Blood pressure, heart rate, percutaneous oxygen saturation (SpO_2_), dyspnea, and fatigue were assessed before and after each ISWT and 6MWT.

**Results:**

A total of 79 healthy participants (mean age = 21.11 ± 1.47 years; 54 females [68.4%] and 25 males [31.6%]) were enrolled, and 74 completed the study. No significant differences were observed between conditions (with vs without a mask) for the 30-second sit-to-stand test, ISWT, or 6MWT distances (*P* > .05). Results revealed a significant main effect of time for all physiological variables (*P* < .001), while no significant condition or interaction effects were found for heart rate or blood pressure during both walking tests (*P* > .05). For the ISWT, a significant Time × Condition interaction was observed for SpO_2_ (*P* = .013) and perceived dyspnea/fatigue (*P* < .05), indicating a greater postexercise decline in SpO_2_ and higher symptomatic perception in the masked condition. Similarly, in the 6MWT, mask use significantly increased perceived dyspnea (*P* < .001), without altering the physiological trajectory.

**Conclusion:**

In healthy young adults, Type I medical mask use does not impair exercise capacity or endurance, despite increasing subjective dyspnea and fatigue. While causing a minor reduction in SpO_2_ during the ISWT, it remains a physiologically safe intervention that should not be considered a barrier to performance in exercise field tests.

## 1. Introduction

The global surge in respiratory illnesses has institutionalized face masks as a standard public health intervention. Medical masks, in particular, serve as a critical barrier against droplet transmission, the primary infection route for numerous respiratory diseases.^[1]^ Given the persistent risk of emerging infectious diseases, mask-wearing is expected to remain a vital component of global health security in current and future public health practice.^[2]^

Face masks are routinely used by certain professions, particularly healthcare workers. During the pandemic period, medical masks were widely adopted, with many individuals wearing them for extended periods while performing activities of varying intensity.^[3]^ Due to the protective role of masks against emerging environmental threats, mask usage has continued to increase even after the pandemic, beyond standard occupational requirements.^[4]^ This sustained use is attributable to the habits established during the pandemic and the masks’ protective role against emerging environmental threats, such as wildfires, earthquakes, and general air pollution.^[4]^

Although the impact of medical masks on the exercise performance of healthy individuals remains incompletely understood, the literature reports conflicting results.^[5,6]^ These inconsistencies in existing studies may stem from the diversity of exercise test protocols, the use of different mask types and brands (including subtypes of surgical masks), and the variability in the age groups tested.^[5,6]^ Consequently, further research is needed to more clearly determine the effects of mask-wearing during physical activity to inform both individual and public health policies.^[6]^ A significant limitation of current research is the reliance on laboratory-based progressive exercise tests, which may not fully reflect real-world conditions.^[5,7,8]^ Most healthy individuals rarely reach such high-intensity activity levels in their daily lives.^[9]^ Preliminary evidence from high-intensity resistance training suggests that mask-wearing does not significantly impair strength-based performance in healthy adults.^[10]^ However, the addition of a spirometric mask over the surgical mask during these progressive tests creates an extra barrier, complicating the generalization of results to everyday life.^[5,8]^ Thus, whether findings from laboratory-based cardiopulmonary exercise protocols can be generalized to functional daily-life activities remains uncertain. Field-based exercise tests may offer a more practical approach for evaluating the effects of mask use during physical activity, as they better reflect the functional demands encountered in daily life. The 6-minute walk test (6MWT) and incremental shuttle walk test (ISWT) are widely used to assess functional exercise capacity under submaximal and progressively increasing workloads, respectively, whereas the 30-second sit-to-stand test (30STS) provides insight into functional lower extremity endurance through repetitive movements commonly performed in everyday activities.^[11–14]^ Unlike laboratory-based exercise protocols, these tests can capture exercise responses under conditions that more closely resemble real-world physical activity. Moreover, evaluating healthy young adults may allow the assessment of mask-related physiological and perceptual responses in the absence of underlying disease-related limitations, providing a clearer understanding of the direct effects of medical mask use during exercise.^[10]^

This study aimed to investigate the effects of a Type I medical (surgical) mask on lower extremity endurance, functional exercise capacity, exercise-related physiological responses (changes in heart rate, blood pressure, and percutaneous oxygen saturation [SpO_2_]), and perceived comfort, defined as the subjective sensation of physical and psychological ease or distress, in healthy young adults in response to the 30STS, ISWT, and 6MWT. We hypothesized that while the use of a Type I medical mask would increase perceived discomfort and sensations of dyspnea, it would not significantly impair exercise performance or alter key physiological responses in healthy young adults.

## 2. Materials and methods

### 2.1. Study design and ethics

This randomized crossover study, conducted between February 10 and November 20, 2021, was approved by the Clinical Research Ethics Committee of Marmara University Faculty of Medicine (approval number: 09.2021.39; approval date: 08.01.2021). Procedures adhered to the Declaration of Helsinki, and all participants provided written informed consent.

### 2.2. Sample size and power analysis

An a priori power analysis^[15]^ – conducted using parameters from similar cardiopulmonary exercise literature due to the limited field-test data at the time of study design – indicated that 79 participants were required to achieve sufficient statistical power. Although 5 participants dropped out during the protocol due to pandemic-related logistical constraints, 74 individuals successfully completed the study. To ensure the reliability of our secondary outcomes, a post hoc power analysis was performed based on the observed effect size (η^2^ = 0.092) for dyspnea scores, which yielded a statistical power of 98.9%. This confirms that our final sample size was more than adequate to detect significant differences in both primary exercise measures and secondary subjective parameters. Healthy adults (18–25 years) were recruited via a convenience sampling method through social media and other communication channels.

Inclusion/exclusion criteria: eligible participants were those without acute/chronic conditions limiting exercise (respiratory, cardiac, or orthopedic) or a recent coronavirus disease-2019 (COVID-19) history. Habitual physical activity levels were not considered a specific criterion for inclusion in this study. Exclusion criteria included facial deformities preventing proper mask fit and mask material allergies.

### 2.3. Interventions and protocol

#### 2.3.1. Study setting and design

The study utilized a within-subject, 2-session crossover design. Tests were performed on 2 separate days – one with a mask and the other without a mask – following an identical testing sequence, with a minimum of 2 days and a maximum of 10 days between sessions (mean = 5 days). To control for potential confounding factors, participants were instructed to avoid strenuous physical activity for 24 hours before each session and to refrain from consuming caffeine or heavy meals for at least 3 hours prior. All evaluations were conducted at similar times of day within the same controlled environment to minimize diurnal variation.

#### 2.3.2. Randomization and mask specification

The sequence of test conditions (mask first vs no-mask first) was randomized a priori using the RAND() function in Microsoft Excel. Each participant was assigned a binary number (0 or 1) based on their unique ID. This procedure was conducted by the researcher responsible for the evaluations. Participants assigned “0” completed the first session without a mask, while those assigned “1” completed the first session with a mask, thus balancing the order of conditions.

A Type I medical mask, which is recommended for public health use, was employed in this study.^[16]^ The mask was constructed of 3 layers of nonwoven spunbond polypropylene fabric and had a documented bacterial filtration efficiency of 95.42%. It featured a flexible elastic ear band and a nose wire to ensure proper facial fit.

#### 2.3.3. Testing protocol

Prior to testing, participants rested unmasked for 15 minutes. Heart rate, SpO_2_, dyspnea, and fatigue were recorded at the end of this period to establish baseline values. Participants then sequentially performed the 30STS, the ISWT, and the 6MWT under the supervision of a physiotherapist.

Unmasked rest intervals of at least 30 minutes were provided between consecutive tests. This specific timeframe was utilized in strict accordance with the American Thoracic Society/European Respiratory Society technical standards^[17]^ to allow physiological parameters (heart rate and SpO_2_) to return to baseline levels and to ensure that performance in subsequent tests was not influenced by residual fatigue from the preceding assessment. Participants were only permitted to proceed to the next test once both SpO_2_ and heart rate had returned to baseline levels.

#### 2.3.4. Primary outcomes

##### 2.3.4.1. Lower extremity endurance

The 30STS was utilized to assess functional lower extremity muscle strength and endurance. Participants were instructed to repeatedly rise from and sit back down on a straight-back chair (approximately 43 cm in height, without armrests) for a duration of 30 seconds. The total number of full stands completed within this time frame was recorded as the primary outcome measure for endurance.^[18]^

##### 2.3.4.2. Exercise capacity and physiological parameters

The ISWT was used to measure maximal exercise capacity, while the 6MWT assessed submaximal exercise capacity. The original ISWT audio recording, obtained directly from the test developers (order number: P769), was employed. The total distance walked during both the ISWT and 6MWT was recorded. Physiological parameters, including heart rate, blood pressure, and SpO_2_, were measured immediately before the start of each test (baseline) and within the first minute following completion (postexercise). Heart rate and SpO_2_ were measured using a pulse oximeter, and single instantaneous scores recorded at these exact time points were used for analysis, while blood pressure was measured using a calibrated digital electronic sphygmomanometer (Braun ExactFit 3, Kaz Europe Sàrl) in a seated position. In addition, subjective perceptions of dyspnea and fatigue were assessed at the same time points using the modified Borg scale (MBS [0–10]) and the visual analog scale (VAS), respectively.^[17]^

#### 2.3.5. Secondary outcomes

##### 2.3.5.1. Identification of limiting factors

At the conclusion of both the ISWT and 6MWT, participants were queried regarding the factor(s) that limited their ability to walk further.^[17]^

##### 2.3.5.2. Mask comfort assessment

Mask comfort was assessed using a 10-item mask comfort questionnaire.^[19]^ This instrument included items related to humidity, heat, breathing resistance, itchiness, tightness, a salty sensation, improper fit, odor, fatigue, and overall discomfort. Each item was rated on a 0 to 10 VAS, where “0” indicated “no discomfort” and “10” indicated “most severe discomfort.” Given that mask use was mandatory in daily life during the study period, the questionnaire was administered under 3 distinct conditions to allow comparison of mask comfort: at rest, during activities of daily living (ADL), and immediately following the ISWT. The scores for the 10 items were summed to calculate total mask comfort scores for the rest, ADL, and ISWT conditions. These total scores were subsequently scaled from 0 (representing the best comfort) to 100 (representing the worst comfort).

### 2.4. Statistical analysis

All analyses were conducted in SPSS 21.0 (IBM Corporation) and Python 3.12 (Pingouin v0.5). The threshold for statistical significance was established at *P* < .05. Normality of the data was assessed using the one-sample Kolmogorov–Smirnov test. Data conforming to a normal distribution are presented as the mean ± standard deviation, whereas non-normally distributed data are expressed as the median and range (minimum–maximum). For physiological parameters with measurements at 2 time points (pre and post) and 2 conditions (without mask and with mask) – specifically heart rate, systolic blood pressure, SpO_2_, dyspnea (VAS and MBS [0–10]), and fatigue (VAS and MBS [0–10]) – a 2 × 2 repeated-measures analysis of variance was applied, with Time (pre vs post) and Condition (without mask vs with mask) as within-subject factors. Partial eta-squared (η²) was calculated as a measure of effect size (small: ≥0.01; medium: ≥0.06; large: ≥0.14). For exercise performance outcomes assessed at a single time point (30STS repetitions, ISWT distance, 6MWT distance), pairwise comparisons between conditions were performed using paired-samples *t* tests for normally distributed variables and the Wilcoxon signed-rank test for non-normally distributed variables. The Friedman test was employed for the 3-level mask comfort comparison (rest, ADL, ISWT), with Bonferroni-corrected post hoc pairwise comparisons. Spearman correlation coefficient was used to evaluate the associations between mask comfort parameters and the changes in dyspnea and fatigue (VAS) during the ISWT and 6MWT. Effect sizes and confidence intervals (CIs) for nonparametric comparisons were calculated using JASP (v. 0.95.4; University of Amsterdam).

## 3. Results

**Figure 1.**
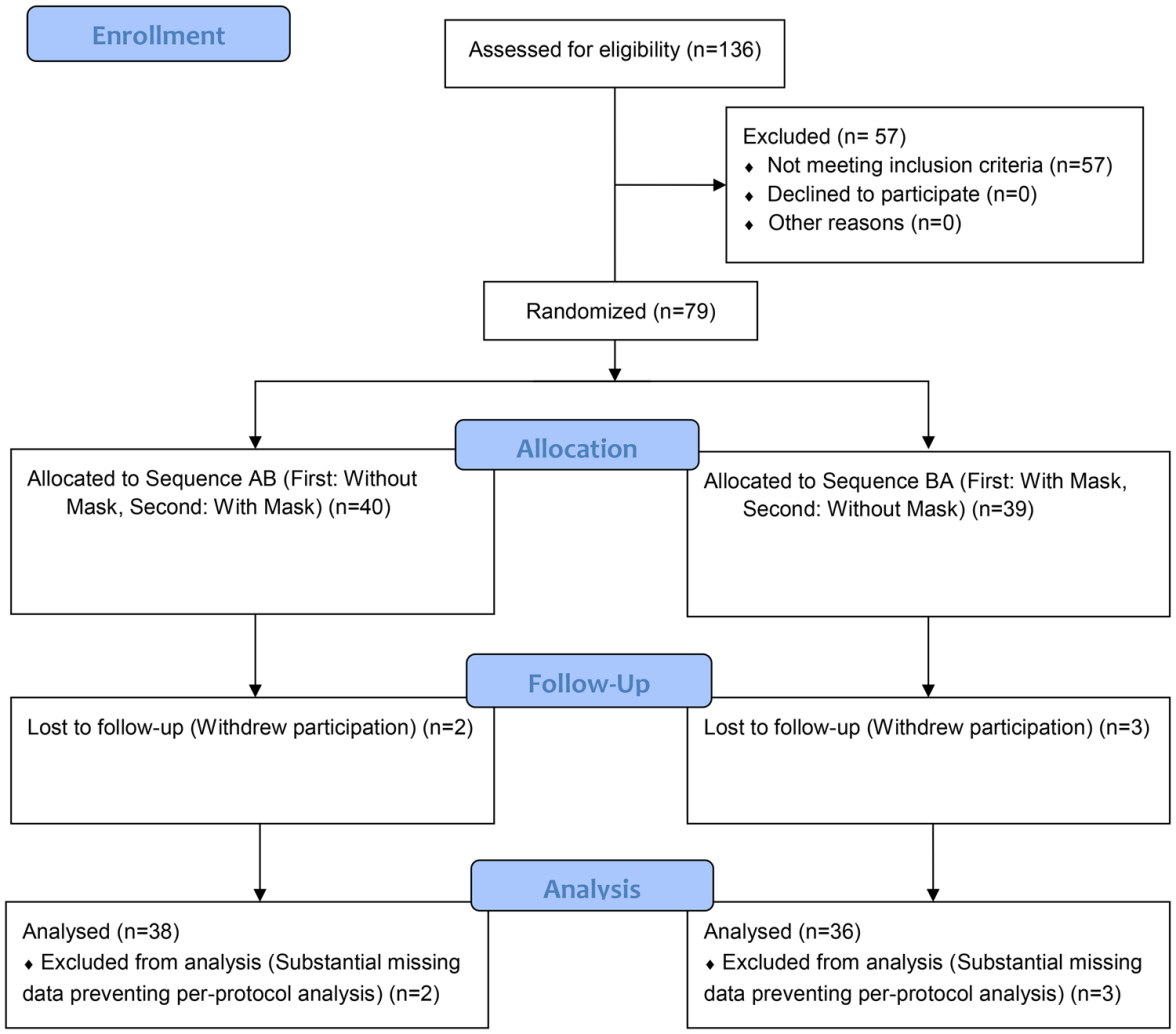
CONSORT flow diagram.

A total of 136 individuals initially volunteered for the study. Following screening, 57 individuals who did not satisfy the inclusion criteria were excluded. Consequently, 79 healthy participants were included in the analysis, consisting of 54 females (68.4%) and 25 males (31.6%). Of the sample included, 5 participants dropped out during the study period, resulting in a final complete dataset of 74 participants. The participant flow throughout the study is detailed in Figure 1.

### 3.1. Participant characteristics

Most participants were nonsmokers (n = 53; 67.1%), followed by current smokers (n = 24; 30.4%), with a small fraction reporting as former smokers (n = 2; 2.5%). In terms of typical mask type, most participants (n = 77; 97.46%) reported using a medical mask. The remaining participants utilized either a cloth mask (n = 1; 1.27%) or a respirator such as an N95 (n = 1; 1.27%). The mean weekly mask use across the entire sample was determined to be 35.12 ± 23.5 hours. Further detailed characteristics are summarized in Table 1.

**Table 1:** Demographic characteristics, smoking history, and mask use of participants (N = 79).

Characteristic	Mean ± SD
Age (yr)	21.11 ± 1.47
Height (m)	1.71 ± 0.09
Body weight (kg)	63.92 ± 13.68
BMI (kg/m^2^)	21.63 ± 2.99
Smoking (pack × yr)	0.43 ± 1.12
Average mask use per week (h)	35.12 ± 23.5
Duration of mask use (mo)	13.53 ± 4.15

The table presents all enrolled participants (N = 79).

BMI = body mass index, SD = standard deviation.

### 3.2. Effects on exercise capacity and physiological responses

No significant differences were observed between conditions (with mask vs without mask) for the 30STS (*t*(73) = 1.052, *P* = .296, Cohen’s *d* = 0.08, 95% CI = −0.15 to 0.30), the ISWT walking distance (*Z* = −1.66, *P* = .097, *R* = 0.23, 95% CI = −0.04 to 0.46), or the 6MWT walking distance (*Z* = −1.91, *P* = .056, *R* = 0.26, 95% CI = −0.00 to 0.48). Full descriptive statistics and test results for exercise performance outcomes are presented in Table 2 (Part A). Physiological parameters were analyzed using a 2 × 2 repeated-measures analysis of variance with Time (pre vs post) and Condition (without mask vs with mask) as within-subject factors. Full results are presented in Table 2 (Part B) and summarized below. ISWT: a significant Time effect was observed for all physiological variables (all *P* < .001), confirming that exercise produced the expected cardiovascular responses irrespective of mask condition. No significant Condition effect was found for heart rate (*F*(1,73) = 0.343, *P* = .560, η² = 0.000), systolic blood pressure (*F*(1,68) = 0.036, *P* = .849, η² < 0.001), or diastolic blood pressure (*F*(1,68) = 0.113, *P* = .738, η² = 0.000), indicating that mask use did not alter cardiovascular responses. SpO_2_ demonstrated a significant Time × Condition interaction (*F*(1,72) = 6.448, *P* = .013, η² = 0.020), with the mask condition associated with a greater postexercise decline in SpO_2_ (with mask: −0.16 ± 0.41%; without mask: 0.00 ± 0.37%), though this extremely small reduction was clinically negligible and remained well within safe physiological limits. A significant Condition effect was found for dyspnea MBS (0–10; *F*(1,44) = 30.833, *P* < .001, η² = 0.092) and fatigue MBS (0–10; *F*(1,49) = 6.528, *P* = .014, η² = 0.022), with higher scores observed throughout the mask condition. Significant Time × Condition interactions were also found for dyspnea MBS (0–10; *F*(1,44) = 5.159, *P* = .028, η² = 0.007) and fatigue MBS (0–10; *F*(1,49) = 6.871, *P* = .012, η² = 0.010), indicating that mask use amplified the exercise-induced rise in perceived dyspnea and fatigue.

**Table 2:** Comparison of 30STS, ISWT, and 6MWT outcomes with and without mask (N = 74).

Part A – Exercise performance outcomes: paired comparison (without mask vs with mask)													
Test	Variable	Without mask				With mask			*P*	Test statistic		Effect size (*r*)	95% CI
		Median		Min/max	Mean ± SD	Median	Min/max	Mean ± SD					
30STS	Repetitions (n)	17		10/28	17.36 ± 4.36	16	10/30	17.01 ± 4.65	.296	*t*(73) = 1.052		*d* = 0.08	−0.15 to 0.30
ISWT	Distance (m)	558.1		348.2/908.0	572.98 ± 114.48	548.0	347.7/928.2	558.85 ± 124.48	.097	*Z* = −1.66		*R* = 0.23	−0.04 to 0.46
6MWT	Distance (m)	615.1		539.3/798.0	629.31 ± 60.06	605.1	505.5/828.0	623.00 ± 66.08	.056	*Z* = −1.91		*R* = 0.26	−0.00 to 0.48

Part B – Physiological parameters: 2 × 2 repeated-measures ANOVA (Time × Condition)													
Test	Variable		Mean ± SD				Time effect		Condition effect		Time × Condition interaction		
			NM pre	NM post	M pre	M post	*F*(1,df)	*P*	*F*(1,df)	*P*	*F*(1,df)	*P*	η2
ISWT	HR (beats/min)		78.0 ± 11.6	119.9 ± 23.2	78.1 ± 10.7	118.4 ± 22.1	*F*(1,73) = 377.417	**<.001**	*F*(1,73) = 0.343	.560	*F*(1,73) = 0.656	.421	0.001
ISWT	SpO_2_ (%)		98.9 ± 0.3	98.9 ± 0.3	99.0 ± 0.1	98.8 ± 0.4	*F*(1,72) = 6.448	**.013**	*F*(1,72) = 0.000	1.000	*F*(1,72) = 6.448	**.013**	0.020
ISWT	Systolic BP (mm Hg)		104.4 ± 13.5	134.0 ± 15.6	104.2 ± 13.5	134.7 ± 17.6	*F*(1,68) = 564.066	**<.001**	*F*(1,68) = 0.036	.849	*F*(1,68) = 0.266	.608	<0.001
ISWT	Diastolic BP (mm Hg)		64.0 ± 7.3	71.8 ± 9.1	63.2 ± 6.9	73.0 ± 7.6	*F*(1,68) = 158.659	**<.001**	*F*(1,68) = 0.113	.738	*F*(1,68) = 3.419	.069	0.004
ISWT	Dyspnea MBS		0.4 ± 0.9	3.0 ± 2.1	1.3 ± 1.9	4.5 ± 2.3	*F*(1,44) = 110.164	**<.001**	*F*(1,44) = 30.833	**<.001**	*F*(1,44) = 5.159	**.028**	0.007
ISWT	Fatigue MBS		1.4 ± 1.3	3.3 ± 1.9	1.6 ± 1.7	4.2 ± 2.1	*F*(1,49) = 98.817	**<.001**	*F*(1,49) = 6.528	**.014**	*F*(1,49) = 6.871	**.012**	0.010
6MWT	HR (beats/min)		77.4 ± 11.5	110.1 ± 20.5	77.3 ± 10.7	109.0 ± 19.5	*F*(1,73) = 307.423	**<.001**	*F*(1,73) = 0.316	.576	*F*(1,73) = 0.403	.527	<0.001
6MWT	SpO_2_ (%)		98.9 ± 0.3	98.9 ± 0.3	98.9 ± 0.3	98.8 ± 0.4	*F*(1,73) = 0.860	.357	*F*(1,73) = 0.755	.388	*F*(1,73) = 1.194	.278	0.003
6MWT	Systolic BP (mm Hg)		102.6 ± 12.6	126.3 ± 16.4	102.7 ± 12.3	125.5 ± 14.1	*F*(1,68) = 325.622	**<.001**	*F*(1,68) = 0.116	.734	*F*(1,68) = 0.340	.562	<0.001
6MWT	Diastolic BP (mm Hg)		63.4 ± 7.8	70.4 ± 7.7	63.6 ± 6.5	70.1 ± 7.7	*F*(1,68) = 129.330	**<.001**	*F*(1,68) = 0.016	.900	*F*(1,68) = 0.244	.623	<0.001
6MWT	Dyspnea MBS		0.4 ± 0.8	2.8 ± 2.0	1.0 ± 1.5	4.3 ± 2.4	*F*(1,48) = 137.743	**<.001**	*F*(1,48) = 25.762	**<.001**	*F*(1,48) = 13.364	**<.001**	0.020
6MWT	Fatigue MBS		1.3 ± 1.4	3.4 ± 1.8	1.8 ± 1.7	4.1 ± 2.3	*F*(1,45) = 94.376	**<.001**	*F*(1,45) = 11.246	**.002**	*F*(1,45) = 2.107	.154	0.001

Part A reports exercise performance outcomes (single time point) compared with the Wilcoxon signed-rank test or paired *t* test. Part B reports physiological parameters with pre/post measurements using 2 × 2 repeated-measures ANOVA (Time × Condition). Bold values (and green shading) indicate *P* < .05. Condition effect = without mask versus with mask; Time × Condition = Interaction.

30STS = 30-second sit-to-stand test, 6MWT = 6-minute walk test, ANOVA = analysis of variance, BP = blood pressure, CI = confidence interval, *d* = Cohen’s *d*, HR = heart rate, ISWT = incremental shuttle walk test, M = with mask, MBS = modified Borg scale, NM = no mask, *r* = effect size (Wilcoxon), SD = standard deviation, SpO_2_ = percutaneous oxygen saturation, η² = partial eta-squared (small ≥ 0.01, medium ≥ 0.06, large ≥ 0.14).

6MWT: the Time effect was again significant for all variables (all *P* < .001), except SpO_2_ (*F*(1,73) = 0.860, *P* = .357). No significant Condition or interaction effects were found for heart rate (Condition: *F*(1,73) = 0.316, *P* = .576; Interaction: *F*(1,73) = 0.403, *P* = .527), systolic blood pressure (Condition: *F*(1,68) = 0.116, *P* = .734; Interaction: *F*(1,68) = 0.340, *P* = .562), diastolic blood pressure (Condition: *F*(1,68) = 0.016, *P* = .900; Interaction: *F*(1,68) = 0.244, *P* = .623), or SpO_2_ (Condition: *F*(1,73) = 0.755, *P* = .388; Interaction: *F*(1,73) = 1.194, *P* = .278). A significant Condition effect was found for dyspnea MBS (0–10; *F*(1,48) = 25.762, *P* < .001, η² = 0.088) and fatigue MBS (0–10; *F*(1,45) = 11.246, *P* = .002, η² = 0.024). The Time × Condition interaction was significant for dyspnea MBS (0–10; *F*(1,48) = 13.364, *P* < .001, η² = 0.020) but not for fatigue MBS (0–10; *F*(1,45) = 2.107, *P* = .154).

### 3.3. Secondary outcomes

#### 3.3.1. Limiting factors, comfort levels, and correlational findings

**Figure 2.**
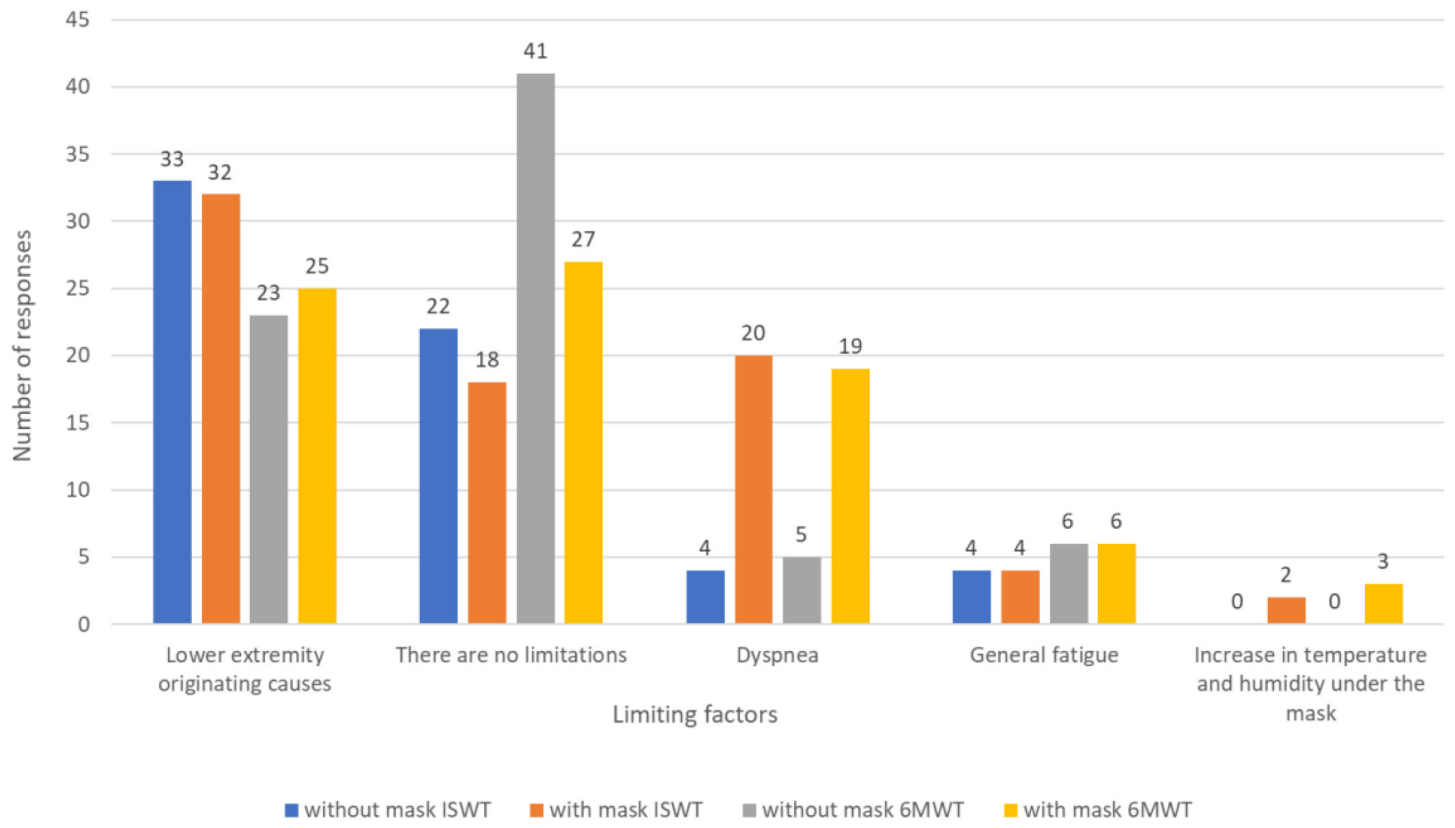
Representation of the primary factors limiting further walking, based on the number of responses. 6MWT = 6-minute walk test, ISWT = incremental shuttle walk test.

Lower extremity discomfort was identified as the primary limiting factor across all test conditions (ISWT and 6MWT, with a mask and without a mask). This discomfort was reported by a substantial portion of the participants, ranging from 29% to 44%. Detailed information on limiting factors is presented in Figure 2.

The participants’ comfort levels were subsequently compared across the 3 effort intensities: rest, ADL, and the post-ISWT condition. A significant difference was observed between the groups for all mask comfort items (*P* < .05). Subsequent pairwise comparisons indicated significant differences between the rest–ADL and rest–ISWT conditions. Mask comfort levels at different effort intensities are presented in Table 3.

**Table 3:** Perceived mask comfort at rest, during activities of daily living, and during the ISWT.

Mask comfort item	Rest min/max	ADL min/max	ISWT min/max	Overall* *P* (Friedman)	Post hoc† *P* values R–A/R–I/A–I
Humid	0–9.5	0–10.0	0–10.0	**<.001**	**<.001**/**<.001**/>.05
Hot	0–9.1	0.8–10.0	0–10.0	**<.001**	**<.001**/**<.001**/>.05
Breath resistance	0–10.0	0–10.0	0.7–10.0	**<.001**	**<.001**/**<.001**/**.001**
Itchy	0–9.4	0–10.0	0–10.0	**<.001**	**<.001**/**<.001**/>.05
Tight	0–9.0	0–9.6	0–10.0	**<.001**	**<.001**/**.017**‡/>.05
Salty	0–10.0	0–10.0	0–10.0	**<.001**	**<.001**/**<.001**/>.05
Unfit	0–9.5	0–10.0	0–10.0	**<.001**	**<.001**/**<.001**/>.05
Odor	0–10.0	0–10.0	0–10.0	**<.001**	**<.001**/**.001**/>.05
Fatigue	0–10.0	0–10.0	0–10.0	**<.001**	**<.001**/**<.001**/**.008**
Overall discomfort	0–10.0	0–10.0	0–10.0	**<.001**	**<.001**/**<.001**/>.05
Total mask comfort score	0–78.0	3.7–86.0	2.0–94.4	**<.001**	**<.001**/**<.001**/>.05

All comfort items were assessed using a 0 to 10 visual analog scale. Post hoc *P* values are reported for 3 pairwise comparisons: rest–ADL (R–A), rest–ISWT (R–I), and ADL–ISWT (A–I), with Bonferroni correction. Bold values (and green shading) indicate *P* < .05.

ADL = activities of daily living, ISWT = incremental shuttle walk test.

* *P*: Friedman test (overall, 3 time points).

† *P*: Post hoc Bonferroni-corrected pairwise comparisons.

‡ Not significant after Bonferroni correction.

Furthermore, the relationships between the functional test results (30STS, ISWT distance, and 6MWT distance) and mask comfort levels were examined. No significant correlations were found between them with a mask, 30STS, ISWT distance, 6MWT results, and comfort items assessed at rest or post-ISWT (*P* > .05). However, the 6MWT distance demonstrated a weak negative correlation with 2 ADL comfort items: “humid” (rs = −0.302, *P* = .008) and “hot” (rs = −0.251, *P* = .028). Weak correlations were also observed between changes in VAS dyspnea and VAS fatigue and certain comfort parameters during the with a mask ISWT and 6MWT (File S1, Supplemental Digital Content 1). Finally, no significant correlations were found between exercise capacity, smoking status, and habitual duration of mask use (*P* > .05).

No harms or unintended effects were reported during the exercise tests with or without masks.

## 4. Discussion

The present study aimed to investigate the effects of Type I medical mask use on both exercise capacity and lower extremity endurance in healthy young adults. The principal finding indicates that wearing a Type I medical mask did not significantly impair either maximal or submaximal exercise capacity, nor did it affect lower extremity endurance. Specifically, the lack of a significant Group × Time interaction for most physiological parameters – with the exception of a minor but statistically significant decline in SpO_2_ during the ISWT – confirms that surgical masks do not meaningfully alter the physiological response of exercise. Conversely, mask use exerted a detrimental impact on subjective parameters, specifically increasing perceived dyspnea, fatigue, and overall discomfort. These findings collectively suggest that while Type I medical masks are well-tolerated physiologically during short-duration, high-intensity exercise and submaximal effort – as evidenced by the negligible effect sizes (η²) observed for the mask condition – their use may be limited by individual subjective tolerance and perceived effort.

The time of this study, mask use was mandatory in nonresidential settings across Turkey. Consequently, participants had been wearing face masks for an average of 13 months due to the pandemic, reporting an average weekly usage of 35 hours. This extended exposure may be a differentiating factor compared to other studies. For instance, Matusiak et al^[3]^ reported significantly shorter mask-wearing durations, a difference likely attributable to variations in national mask mandates and cultural norms.

Our finding that mask use did not impair functional exercise performance is consistent with the limited existing literature. In particular, previous studies investigating the effects of face masks on exercise performance in healthy young adults have reported no detrimental effects on muscular performance during high-intensity resistance training sessions.^[10]^ Although resistance-based protocols differ from ambulatory field tests in terms of metabolic and biomechanical demands, this evidence is particularly relevant to our 30STS findings, which primarily reflect functional lower extremity strength and endurance. Taken together, these results suggest that the use of a Type I medical mask does not impose a meaningful mechanical or physiological constraint capable of limiting short-duration explosive movements or sustained functional tasks in healthy young adults. In the present study, functional exercise performance was evaluated using 3 complementary and widely validated field tests to capture distinct dimensions of physical function under conditions that more closely reflect real-world activity. The 6MWT assesses submaximal functional exercise capacity and is representative of ADL, whereas the ISWT provides an externally paced, progressively increasing workload and has been shown to yield outcomes comparable to cardiopulmonary exercise testing (CPET) for estimating maximal exercise capacity in healthy individuals.^[20,21]^ Although CPET remains the reference standard for maximal exercise assessment, its cost and limited accessibility have led to increased reliance on field-based alternatives such as the ISWT in applied research settings. The 30STS, in contrast, evaluates functional lower limb endurance through repeated sit-to-stand transitions that are frequently encountered in daily life and rehabilitation contexts.^[11–14]^ Collectively, these field tests offer an accessible, cost-effective, and ecologically valid assessment framework that extends beyond laboratory-based ergometry and enhances the translational relevance of the present findings.

Recent systematic reviews and meta-analyses^[5,22]^ support our findings, indicating that mask use has little to no impact on exercise performance or cardiac parameters. The discrepancy between our findings and those of studies reporting an effect of mask use on exercise capacity may be attributed to differences in participants’ levels of physical activity. In athletes, higher muscular fitness may allow them to reach greater exercise intensities without muscle-related limitations, enabling a more direct assessment of the mask’s effect. Conversely, it is well documented that physical activity levels in young adults decreased during the pandemic.^[23]^ In the present study, participants were healthy young adults rather than professional athletes, and they were specifically selected to enable the isolated evaluation of mask-related physiological and perceptual responses without the confounding effects of underlying cardiopulmonary, neuromuscular, or metabolic disease. Moreover, this population represented individuals who commonly engage in educational, occupational, and recreational activities while wearing medical masks for prolonged periods. While we did not formally quantify habitual physical activity at baseline, it is highly probable that their habitual physical fitness levels influenced the observed performance. Specifically, the lower activity levels typically seen in such cohorts may have led to earlier performance limitations due to muscular factors rather than respiratory constraints imposed by the mask. This is further supported by our observation that the ISWT is a symptom-limited test, and many participants reported muscular fatigue as the primary factor restricting test continuation. Wearing a spirometric mask over a medical mask significantly decreases the space between the mask and the wearer’s face, which can make breathing more difficult during exertion and limit the generalizability of the findings.^[8]^ In routine daily use, however, individuals wear only the medical mask adjusted to the face without additional respiratory apparatus. By evaluating mask use under conditions that closely mimic typical daily wear, this study provides high ecological validity, making the findings more directly applicable to standard clinical and community settings.

Person et al^[24]^ conducted a study involving 44 healthy, nonsmoking subjects (26 females, 18 males; mean age = 21.6 ± 2.8 years) and reported 6MWT results similar to those of our study. In addition, Cabanillas-Barea et al^[25]^ evaluated 50 healthy volunteers (26 males, 24 females; mean age = 20.96 ± 5.36 years) and found that medical masks increased perceived shortness of breath but did not affect the activity of the middle scalene and sternocleidomastoid muscles, corroborating the findings of Person et al.^[24]^ In contrast, Dirol et al^[26]^ conducted a study with 100 healthy nonsmoking volunteers (58 females, 42 males; mean age = 40.87 ± 12.73 years) and reported that mask use led to greater increases in respiratory rate and heart rate, as well as a significant decrease in 6MWT distance (14.5 m) and SpO_2_ compared to the no-mask condition. Notably, within their cohort, 60% of the participants were in the 35 to 65 age subgroup (n = 60, mean age = 49.70 years). The differences between their results and ours may be attributed to the younger age of our participants. Nevertheless, our 6MWT results approached statistical significance. However, this difference does not translate into a clinically significant reduction in exercise performance, as the official American Thoracic Society/European Respiratory Society technical standards suggest a minimal important difference of 30 m for the 6MWT in adults with chronic respiratory disease.^[17]^ Since the changes observed in our study and similar literature (e.g., 14.5 m in Dirol et al) fall below this 30-m threshold, these findings indicate that while statistical variations may occur, the use of a surgical mask does not lead to a clinically meaningful impairment in functional walking capacity. Given the varying findings in the literature, further investigation is warranted.

Findings reported in the literature regarding mask use are often linked to reductions in pulmonary function.^[5,15,25,27–31]^ Lässing et al reported that airway resistance, as measured by body plethysmography, nearly doubled in healthy young adults following medical mask use.^[32]^ It has been reported that increased airway resistance reduces the concentration of inhaled oxygen and raises the workload of respiratory muscles, thereby increasing oxygen consumption.^[26,31]^ The notion that wearing a medical mask elevates respiratory effort in parallel with increasing activity intensity, particularly during rest, is supported by previous studies.^[26,31]^ On the other hand, the effects of the additional dead space created around the nose and mouth by a medical mask have been highlighted,^[26,33]^ with re-inhalation of accumulated CO_2_ potentially leading to hypercapnia.^[33,34]^

We observed that mask use led to a 1% decrease in SpO_2_ during the ISWT. A systematic review and meta-analysis reported that medical masks may slightly reduce oxygen saturation during maximal exercise but not during submaximal exercise.^[22]^ In contrast, a 4% decrease in SpO_2_ has been previously reported in healthy individuals during the ISWT, independent of mask use.^[35]^ Although our results revealed a significant Time × Condition interaction for SpO_2_ (*P* = .013), the observed 1% decline is clinically negligible and falls well below the threshold of clinical significance. Furthermore, this minor variation did not translate into a reduction in walking distance, suggesting that the slight oxygen desaturation did not impose a functional limitation.

Neural mechanisms underlying dyspnea are largely attributed to corollary discharge. Dyspnea arises from a mismatch between afferent sensory signals reaching higher brain centers and the corollary discharge associated with motor commands. At this stage, it has been suggested that wearing a face mask increases breathing resistance, enhancing afferent input from the muscle spindles of the respiratory muscles and creating a mismatch between the mechanical sensation of respiration and the corollary discharge of the motor command.^[36]^ A recent study demonstrated that the “air hunger” component of dyspnea is particularly pronounced during exercise performed with a mask, supporting the mismatch theory.^[37]^ Moisture accumulation in medical masks has also been reported to contribute to increased breathing resistance,^[38]^ while psychological factors associated with mask use may further exacerbate dyspnea.^[36]^

Comfort levels were assessed at rest, during ADL, and at the end of the ISWT. Humidity, heat, and breath resistance were the parameters associated with the highest reported discomfort. Similar findings have been reported previously in the literature.^[19,33]^ The medical mask obstructs evaporation and convection pathways on the covered areas of the face, trapping exhaled warm air and creating a microclimate with elevated temperature and humidity.^[39]^ Zhang et al^[40]^ demonstrated that participants perceived increased warmth when wearing a medical mask. This effect may be related to the cheek area, which is partially covered by the mask and is 2 to 3 times more sensitive to thermal stimuli than the body average.^[41]^ Considering that increased humidity and thermal stress may add to breathing resistance, the interrelationship between temperature, humidity, and breath resistance is evident.^[19,38]^ Excessive increases in humidity and temperature are important not only for comfort but also because they may compromise the filtration efficiency of the mask.^[42]^ Mask comfort is also critical for ensuring compliance with mask use.^[26,39]^ The contribution of mask humidity to increased breathing resistance,^[38]^ along with the notion that elevated facial temperature and humidity may exacerbate the perception of dyspnea,^[43]^ highlights the importance of evaluating and improving mask comfort, particularly for populations beyond young healthy adults, such as older individuals or patients with chronic respiratory conditions. Our findings, together with published literature, confirm the presence of mask-related challenges and support the need for the development of improved mask designs. The significantly greater dyspnea and fatigue reported during mask-wearing suggest that while these neural and thermal mechanisms amplify the perception of effort, they do not reach a threshold that impairs functional performance in this cohort.

Egger et al^[44]^ reported that, in well-trained athletes, 69% of participants indicated that the vacuum effect caused by increased moisture and deformation of the medical mask contributed to exercise termination by inducing acute dyspnea. In the present study, although the frequency of potential mask-induced dyspnea as a limiting factor increased, it did not reach a level sufficient to cause a statistically significant reduction in exercise capacity. Notably, lower extremity-related factors were the primary limiting cause in both without mask and with mask ISWT and 6MWT sessions. These limitations were primarily muscular, especially involving the tibialis anterior, with participants reporting burning, contraction, pain, and fatigue. Similar symptoms were reported in the gastrocnemius and soleus muscles, though less frequently. Previous studies have indicated that wearing a medical mask does not adversely affect muscle oxygenation.^[8,45]^ Therefore, we estimate that reduced muscular fitness was the main factor limiting exercise capacity in our participants.

This study has certain limitations. Although we focused on acute effects and excluded individuals with a recent COVID-19 history or suspected COVID-19, the possibility of asymptomatic infection at the time of testing remains a residual risk that could not be entirely ruled out. A particularly important limitation concerns the characterization of the study sample; participants’ habitual physical activity levels were not formally assessed. This omission means we were unable to quantify the baseline physical fitness heterogeneity within the cohort, and therefore, we cannot definitively determine how this potential confounding factor may have influenced the performance results observed during the exercise tests. Furthermore, the selected functional field tests predominantly represent low- to moderate-intensity exercise. Although the ISWT provides a progressively increasing workload and may induce near-maximal exertion in some participants, the findings may not be generalizable to vigorous or sustained high-intensity exercise conditions. During higher-intensity exercise, increased ventilatory demand may amplify subjective symptoms such as dyspnea, breathing resistance, and discomfort associated with mask use. Consequently, this prevents the direct generalization of our findings to more vigorous physical activities or athletic performance contexts. Future studies involving larger populations and incorporating CPET or high-intensity exercise protocols may further clarify the physiological and perceptual effects of surgical masks during vigorous exertion in individuals with different activity levels.

In conclusion, the results of this study demonstrate that the use of a Type I medical mask in healthy young adults does not significantly impact objective measures such as maximal or submaximal exercise capacity or lower extremity endurance. Our aggregate results for most physiological parameters confirm that surgical masks do not compromise the physiological trajectory of exercise performance. While a statistically significant but clinically negligible decline in SpO_2_ was observed during the ISWT, it did not translate into functional impairment. However, a key finding is that mask use negatively affects subjective parameters, leading to an increased perception of dyspnea and fatigue. Our findings also highlight that the physical discomfort associated with mask use is notable and intensifies significantly with increased physical effort.

Given the persistent threat of emerging infectious diseases, mask use will remain a primary protective public health measure. The pronounced discomfort observed in this age group, however, poses a potential barrier to compliance. Therefore, future efforts should focus on developing new mask designs that significantly enhance user comfort, particularly during physical activity. Furthermore, research must continue to investigate the multidimensional effects of mask use, including long-term psychological and physical impacts, to optimize public health strategies.

## Author contributions

**Conceptualization:** Meryem Bektaş Karakuş, Aysel Yildiz Özer.

**Data curation:** Meryem Bektaş Karakuş.

**Formal analysis:** Meryem Bektaş Karakuş, Eren Timurtaş, Aysel Yildiz Özer.

**Investigation:** Meryem Bektaş Karakuş, Aysel Yildiz Özer.

**Methodology:** Meryem Bektaş Karakuş, Eren Timurtaş, Aysel Yildiz Özer.

**Project administration:** Meryem Bektaş Karakuş, Aysel Yildiz Özer.

**Resources:** Meryem Bektaş Karakuş, Aysel Yildiz Özer.

**Visualization:** Meryem Bektaş Karakuş, Aysel Yildiz Özer.

**Validation:** Eren Timurtaş.

**Writing – original draft:** Meryem Bektaş Karakuş, Aysel Yildiz Özer.

**Writing – review & editing:** Meryem Bektaş Karakuş, Eren Timurtaş, Aysel Yildiz Özer.



## References

[1] Asadi S Cappa CD Barreda S Wexler AS Bouvier NM Ristenpart WD. Efficacy of masks and face coverings in controlling outward aerosol particle emission from expiratory activities. Sci Rep. 2020;10:15665. 32973285 10.1038/s41598-020-72798-7 PMC7518250

[2] Baker RE Mahmud AS Miller IF. Infectious disease in an era of global change. Nat Rev Microbiol. 2022;20:193–205. 34646006 10.1038/s41579-021-00639-z PMC8513385

[3] Matusiak L Szepietowska M Krajewski PK Białynicki-Birula R Szepietowski JC. The use of face masks during the COVID-19 pandemic in Poland: a survey study of 2315 young adults. Dermatol Ther. 2020;33:e13909. 32602208 10.1111/dth.13909 PMC7361243

[4] Laumbach RJ Cromar KR. Personal interventions to reduce exposure to outdoor air pollution. Annu Rev Public Health. 2022;43:293–309. 34936825 10.1146/annurev-publhealth-052120-103607

[5] Zheng C Poon ET Wan K Dai Z Wong SH. Effects of wearing a mask during exercise on physiological and psychological outcomes in healthy individuals: a systematic review and meta-analysis. Sports Med. 2023;53:125–50. 36001290 10.1007/s40279-022-01746-4 PMC9400006

[6] Farah W Abusalih MF Hasan B. Safety implications of mask use: a systematic review and evidence map. BMJ Evid Based Med. 2025;30:91–103. 10.1136/bmjebm-2024-113028 PMC12013569 39326926

[7] Ramoti N Siahaan AM Indharty S Adella CA. Effect of face masks on dyspnea perception, cardiopulmonary parameters, and facial temperature in healthy adults. Narra J. 2024;4:e574. 38798847 10.52225/narra.v4i1.574 PMC11125298

[8] Shaw K Butcher S Ko J Zello GA Chilibeck PD. Wearing of cloth or disposable surgical face masks has no effect on vigorous exercise performance in healthy individuals. Int J Environ Res Public Health. 2020;17:8110. 33153145 10.3390/ijerph17218110 PMC7662944

[9] Troiano RP Berrigan D Dodd KW Mâsse LC Tilert T McDowell M. Physical activity in the United States measured by accelerometer. Med Sci Sports Exerc. 2008;40:181–8. 18091006 10.1249/mss.0b013e31815a51b3

[10] Rial-Vázquez J Nine I Guerrero-Moreno JM. Face masks at the gymnasium: physiological responses and mechanical performance are not compromised by wearing surgical or filtering facepiece 2 masks in healthy subjects. J Strength Cond Res. 2023;37:1404–10. 37347944 10.1519/JSC.0000000000004401

[11] Singh SJ Puhan MA Andrianopoulos V. An official systematic review of the European Respiratory Society/American Thoracic Society: measurement properties of field walking tests in chronic respiratory disease. Eur Respir J. 2014;44:1447–78. 25359356 10.1183/09031936.00150414

[12] Ronai P Mendola N. The incremental shuttle walk test. ACSM’S Health Fitness J. 2021;25:42–7.

[13] Baldwin MM Daynes E Karsanji U. The safety, physiological response and repeatability of the incremental shuttle walk test in survivors of COVID-19. ERJ Open Res. 2025;11:00089–2025. 41403426 10.1183/23120541.00089-2025 PMC12704175

[14] Reychler G Straeten CV Schalkwijk A Poncin W. Effects of surgical and cloth facemasks during a submaximal exercise test in healthy adults. Respir Med. 2021;186:106530. 34273733 10.1016/j.rmed.2021.106530 PMC8452602

[15] Fikenzer S Uhe T Lavall D. Effects of surgical and FFP2/N95 face masks on cardiopulmonary exercise capacity. Clin Res Cardiol. 2020;109:1522–30. 32632523 10.1007/s00392-020-01704-y PMC7338098

[16] Turkish Standards Institution. Medical face masks – requirements and test methods. 2019. Available at: https://intweb.tse.org.tr/Standard/Standard/Standard.aspx?081118051115108051104119110104055047105102120088111043113104073090118115076071111050106050106055. Accessed December 15, 2021.

[17] Holland AE Spruit MA Troosters T. An official European Respiratory Society/American Thoracic Society technical standard: field walking tests in chronic respiratory disease. Eur Respir J. 2014;44:1428–46. 25359355 10.1183/09031936.00150314

[18] Jones CJ Rikli RE Beam WC. A 30-s chair-stand test as a measure of lower body strength in community-residing older adults. Res Q Exerc Sport. 1999;70:113–9. 10380242 10.1080/02701367.1999.10608028

[19] Li Y Tokura H Guo YP. Effects of wearing N95 and surgical facemasks on heart rate, thermal stress and subjective sensations. Int Arch Occup Environ Health. 2005;78:501–9. 15918037 10.1007/s00420-004-0584-4 PMC7087880

[20] Lima LP Leite HR Matos MA. Cardiorespiratory fitness assessment and prediction of peak oxygen consumption by incremental shuttle walking test in healthy women. PLoS One. 2019;14:e0211327. 30730949 10.1371/journal.pone.0211327 PMC6366724

[21] Neves CDC Lacerda ACR Lage VKS. Cardiorespiratory responses and prediction of peak oxygen uptake during the shuttle walking test in healthy sedentary adult men. PLoS One. 2015;10:e0117563. 25659094 10.1371/journal.pone.0117563 PMC4319837

[22] Shaw KA Zello GA Butcher SJ Ko JB Bertrand L Chilibeck PD. The impact of face masks on performance and physiological outcomes during exercise: a systematic review and meta-analysis. Appl Physiol Nutr Metab. 2021;46:693–703. 33901405 10.1139/apnm-2021-0143

[23] Alaca N Koçyiğit D Tokgöz S Berikman B. Comparison of anxiety and physical activity levels and physical activity barriers of physiotherapy and rehabilitation department students during and before the COVID-19 pandemic. J Health Pro Res. 2022;4:12–26.

[24] Person E Lemercier C Royer A Reychler G. [Effect of a surgical mask on six minute walking distance]. Rev Mal Respir. 2018;35:264–8. 29395560 10.1016/j.rmr.2017.01.010

[25] Cabanillas-Barea S Rodríguez-Sanz J Carrasco-Uribarren A. Effects of using the surgical mask and FFP2 during the 6-min walking test. A randomized controlled trial. Int J Environ Res Public Health. 2021;18:12420. 34886145 10.3390/ijerph182312420 PMC8656790

[26] Dirol H Alkan E Sindel M Ozdemir T Erbas D. The physiological and disturbing effects of surgical face masks in the COVID-19 era. Bratisl Lek Listy. 2021;122:821–5. 34672675 10.4149/BLL_2021_131

[27] Alkan B Ozalevli S Akkoyun Sert O. Maximal exercise outcomes with a face mask: the effects of gender and age differences on cardiorespiratory responses. Ir J Med Sci. 2022;191:2231–7. 34837141 10.1007/s11845-021-02861-3 PMC8625666

[28] Epstein D Korytny A Isenberg Y. Return to training in the COVID-19 era: the physiological effects of face masks during exercise. Scand J Med Sci Sports. 2021;31:70–5. 32969531 10.1111/sms.13832 PMC7646657

[29] Fukushi I Nakamura M Kuwana SI. Effects of wearing facemasks on the sensation of exertional dyspnea and exercise capacity in healthy subjects. PLoS One. 2021;16:e0258104. 34591935 10.1371/journal.pone.0258104 PMC8483295

[30] Jesus JP Gomes M Dias-Gonçalves A Correia JM Pezarat-Correia P Mendonca GV. Effects of surgical masks on the responses to constant work-rate cycling performed at different intensity domains. Clin Physiol Funct Imaging. 2022;42:43–52. 34753208 10.1111/cpf.12734 PMC8646879

[31] Zhang G Li M Zheng M. Effect of surgical masks on cardiopulmonary function in healthy young subjects: a crossover study. Front Physiol. 2021;12:710573. 34566679 10.3389/fphys.2021.710573 PMC8461071

[32] Lässing J Falz R Pökel C. Effects of surgical face masks on cardiopulmonary parameters during steady state exercise. Sci Rep. 2020;10:22363. 33349641 10.1038/s41598-020-78643-1 PMC7752911

[33] Kisielinski K Giboni P Prescher A. Is a mask that covers the mouth and nose free from undesirable side effects in everyday use and free of potential hazards? Int J Environ Res Public Health. 2021;18:4344. 33923935 10.3390/ijerph18084344 PMC8072811

[34] Atangana E Atangana A. Facemasks simple but powerful weapons to protect against COVID-19 spread: can they have sides effects? Results Phys. 2020;19:103425. 33014697 10.1016/j.rinp.2020.103425 PMC7525365

[35] Seixas DM Seixas DMT Pereira MC Moreira MM Paschoal IA. Oxygen desaturation in healthy subjects undergoing the incremental shuttle walk test. J Bras Pneumol. 2013;39:440–6. 24068265 10.1590/S1806-37132013000400007 PMC4075871

[36] Fukushi I Pokorski M Okada Y. Mechanisms underlying the sensation of dyspnea. Respir Investig. 2021;59:66–80. 10.1016/j.resinv.2020.10.007 33277231

[37] Ferguson ON Mitchell RA Schaeffer MR. Effects of face masks on the multiple dimensions and neurophysiological mechanisms of exertional dyspnea. Med Sci Sports Exerc. 2023;55:450–61. 36469484 10.1249/MSS.0000000000003074

[38] Scheid JL Lupien SP Ford GS West SL. Commentary: physiological and psychological impact of face mask usage during the COVID-19 pandemic. Int J Environ Res Public Health. 2020;17:6655. 32932652 10.3390/ijerph17186655 PMC7558090

[39] Roberge RJ Kim JH Benson SM. Absence of consequential changes in physiological, thermal and subjective responses from wearing a surgical mask. Respir Physiol Neurobiol. 2012;181:29–35. 22326638 10.1016/j.resp.2012.01.010

[40] Zhang R Liu J Zhang L Lin J Wu Q. The distorted power of medical surgical masks for changing the human thermal psychology of indoor personnel in summer. Indoor Air. 2021;31:1645–56. 33818847 10.1111/ina.12830 PMC8251099

[41] Luo M Wang Z Zhang H. High-density thermal sensitivity maps of the human body. Build Environ. 2020;167:106435.

[42] Lee KP Yip J Kan CW Chiou JC Yung KF. Reusable face masks as alternative for disposable medical masks: factors that affect their wear-comfort. Int J Environ Res Public Health. 2020;17:6623. 32932918 10.3390/ijerph17186623 PMC7558362

[43] Hopkins SR Dominelli PB Davis CK. Face masks and the cardiorespiratory response to physical activity in health and disease. Ann Am Thorac Soc. 2021;18:399–407. 33196294 10.1513/AnnalsATS.202008-990CME PMC7919154

[44] Egger F Blumenauer D Fischer P. Effects of face masks on performance and cardiorespiratory response in well-trained athletes. Clin Res Cardiol. 2022;111:264–71. 34091726 10.1007/s00392-021-01877-0 PMC8179953

[45] Ahmadian M Ghasemi M Nasrollahi Borujeni N. Does wearing a mask while exercising amid COVID-19 pandemic affect hemodynamic and hematologic function among healthy individuals? Implications of mask modality, sex, and exercise intensity. Phys Sportsmed. 2022;50:257–68. 33902400 10.1080/00913847.2021.1922947

