# Supplementary material for: The pandemic legacy face mask: hidden barrier or insignificant factor in exercise performance? A randomized crossover trial
Source: Medicine (Baltimore). 2026 Aug 14;105(33):e50179. doi: 10.1097/MD.0000000000050179 (PMC13480884; doi:10.1097/MD.0000000000050179)
Supplement: Supplementary file 1 [file medi-105-e50179-s001.docx]

**Supplemental Digital Content 1.** Spearman Correlations Between Mask Comfort Parameters and Changes in Dyspnoea and Fatigue (VAS) During ISWT and 6MWT

*Spearman's rₛ is reported only for statistically significant correlations (p < 0.05). Empty cells indicate p > 0.05 (not significant). Correlations were assessed at three time points: Rest (R), Activities of Daily Living (ADL), and ISWT (I). Green shading indicates p < 0.05.*

| **Additional File 1. Spearman Correlations between Mask Comfort Parameters and Dyspnoea/Fatigue Changes during ISWT and 6MWT** | | | | | | | | | |
| --- | --- | --- | --- | --- | --- | --- | --- | --- | --- |
| **Mask Comfort Item** | **Time Point** | **ISWT — ΔVAS Dyspnoea** | | **ISWT — ΔVAS Fatigue** | | **6MWT — ΔVAS Dyspnoea** | | **6MWT — ΔVAS Fatigue** | |
|  |  | **p** | **rₛ** | **p** | **rₛ** | **p** | **rₛ** | **p** | **rₛ** |
| **Humid** | *Rest* | **0.015** | 0.276 | >0.05 |  | **<0.001** | 0.397 | **0.040** | 0.234 |
|  | *ADL* | **0.008** | 0.301 | >0.05 |  | **0.033** | 0.243 | >0.05 |  |
|  | *ISWT* | **0.013** | 0.283 | >0.05 |  | **<0.001** | 0.395 | **0.007** | 0.307 |
| **Hot** | *Rest* | **0.017** | 0.272 | >0.05 |  | **0.042** | 0.232 | >0.05 |  |
|  | *ADL* | **0.030** | 0.248 | >0.05 |  | >0.05 |  | >0.05 |  |
|  | *ISWT* | **0.006** | 0.311 | >0.05 |  | **0.014** | 0.279 | >0.05 |  |
| **Breath resistance** | *Rest* | >0.05 |  |  | >0.05 | **0.016** | 0.274 | **0.038** | 0.237 |
|  | *ADL* | **0.014** | 0.278 | >0.05 |  | **0.011** | 0.289 | >0.05 |  |
|  | *ISWT* | **0.012** | 0.284 | >0.05 |  | **0.001** | 0.384 | **0.003** | 0.332 |
| **Itchy** | *Rest* | >0.05 |  | >0.05 |  | >0.05 |  | >0.05 |  |
|  | *ADL* | >0.05 |  | >0.05 |  | >0.05 |  | >0.05 |  |
|  | *ISWT* | **0.036** | 0.239 | >0.05 |  | >0.05 |  | >0.05 |  |
| **Tight** | *Rest* | **0.027** | 0.252 | >0.05 |  | >0.05 |  | >0.05 |  |
|  | *ADL* | **0.020** | 0.265 | >0.05 |  | >0.05 |  | >0.05 |  |
|  | *ISWT* | **0.028** | 0.250 | >0.05 |  | >0.05 |  | >0.05 |  |
| **Salty** | *Rest* | **0.009** | 0.297 | **0.035** | 0.241 | >0.05 |  | >0.05 |  |
|  | *ADL* | >0.05 |  | >0.05 |  | >0.05 |  | >0.05 |  |
|  | *ISWT* | >0.05 |  | >0.05 |  | >0.05 |  | >0.05 |  |
| **Unfit** | *Rest* | **0.017** | 0.272 | >0.05 |  | **0.019** | 0.267 | >0.05 |  |
|  | *ADL* | **0.035** | 0.241 | >0.05 |  | **0.009** | 0.295 | >0.05 |  |
|  | *ISWT* | **0.003** | 0.334 | **0.019** | 0.266 | **0.028** | 0.251 | >0.05 |  |
| **Odour** | *Rest* | **0.021** | 0.263 | >0.05 |  | **0.003** | 0.329 | **<0.001** | 0.427 |
|  | *ADL* | >0.05 |  | >0.05 |  | >0.05 |  | **0.001** | 0.368 |
|  | *ISWT* | >0.05 |  | **0.049** | 0.226 | >0.05 |  | >0.05 |  |
| **Fatigue (mask)** | *Rest* | **0.002** | 0.342 | **0.012** | 0.284 | **0.034** | 0.242 | **0.031** | 0.246 |
|  | *ADL* | **0.022** | 0.261 | >0.05 |  | >0.05 |  | >0.05 |  |
|  | *ISWT* | **0.002** | 0.347 | **0.005** | 0.317 | **0.007** | 0.303 | **0.006** | 0.308 |
| **Overall discomfort** | *Rest* | **0.021** | 0.262 | >0.05 |  | 0.050 |  | >0.05 |  |
|  | *ADL* | >0.05 |  | >0.05 |  | >0.05 |  | >0.05 |  |
|  | *ISWT* | **0.008** | 0.300 | >0.05 |  | **0.016** | 0.273 | >0.05 |  |
| **Total mask comfort** | *Rest* | **0.001** | 0.387 | >0.05 |  | **0.001** | 0.369 | **0.016** | 0.274 |
|  | *ADL* | **0.004** | 0.325 | >0.05 |  | >0.05 |  | >0.05 |  |
|  | *ISWT* | **0.001** | 0.376 | **0.011** | 0.288 | **0.025** | 0.256 | **0.028** | 0.251 |

*ISWT: Incremental Shuttle Walk Test; 6MWT: 6-Minute Walk Test; ADL: Activities of Daily Living;*  *VAS: Visual Analogue Scale; rₛ: Spearman's correlation coefficient.*  *Green shading = p < 0.05; grey values = p > 0.05 (not significant).*
